# Stories of experiences of care for growth hormone deficiency: the CRESCERE project

**DOI:** 10.4155/fso.15.82

**Published:** 2016-02-25

**Authors:** Maria G Marini, Paola Chesi, Laura Mazzanti, Laura Guazzarotti, Teresa D Toni, Maria C Salerno, Annunziata Officioso, Maria Parpagnoli, Cristina Angeletti, Maria F Faienza, Maria L Iezzi, Tommaso Aversa, Cinzia Sacchetti

**Affiliations:** 1 ISTUD Foundation, Health Care & Wellbeing Area, Milan, Italy; 2 Paediatric Endocrinology Unit, Paediatric Department ‘Sant'Orsola Malpighi’ University Hospital, Bologna, Italy; 3 Pediatrician Clinic, ‘Luigi Sacco’ Hospital, Milan, Italy; 4 Pediatrician Hospital ‘Gaslini’, Genoa, Italy; 5 Paediatric Section, Department of Translational Medical Sciences ‘Federico II’, Naples, Italy; 6 ‘Meyer’ Children Hospital, Florence, Italy; 7 Paediatric Section, Hospital District Area n. 2, Senigallia, Italy; 8 General Hospital ‘Giovanni XXIII’, Bari, Italy; 9 Pediatrician Clinic, Hospital District ‘San Salvatore’, L'Aquila, Italy; 10 Department of Paediatrics, University of Messina, Messina, Italy; 11 Italian Association of Families with subjects with a GHD A.Fa.D.O.C., Vicenza, Italy

**Keywords:** growth hormone, therapy, Narrative Medicine, illness, story

## Abstract

**Aims:**

Growth hormone deficiency therapy is demanding for patients and caregivers. Teams engaged in the clinical management of growth hormone deficiency therapy need to know how families live with this condition, to provide an adequate support and prevent the risk of withdrawal from therapy.

**Methods:**

Using Narrative Medicine, testimonies from patients, their parents and providers of care were collected from 11 Italian centers. Narrations were analyzed throughout an elaboration of recurring words and expressions.

**Results:**

Although care management and outcomes were considered satisfying in the 182 collected narratives, recurring signals of intolerance among adolescents and the worry of not being well informed about side effects among parents are open issues.

**Conclusion:**

Narratives found that communication issues could decrease adherence and influence the physicians’ clinical practice.

## Abstract (lay abstract)

**Lay abstract:** Using Narrative Medicine, testimonies from children and adolescents with growth hormone deficiency, their parents and providers of care were collected in 11 Italian Centers. Although the general high level of satisfaction for the results of the treatment, the analysis of the narrations revealed that adolescents can show recurring signals of intolerance and parents can feel worried and not well informed about side effects. Communication issues could decrease the adherence to therapy, and teams engaged in the clinical management of growth hormone deficiency need to strengthen their communication with all the family members, to provide an adequate support and prevent the risk of withdrawal from therapy.

## Growth hormone therapy

The recombinant human growth hormone (GH) therapy has been shown to significantly improve height outcome in patient with GH deficiency (GHD) [1]. Throughout a long-term and daily administration of GH, height velocity increases and final height may reach its genetic target [2]. Subcutaneous daily injections with different devices are currently the route of choice for GH treatment [3,4]. Because of this schedule, therapy is challenging to follow. The use of a needle might represent a daily and painful trouble for children, and the requirements for reconstitution of the freeze-dried preparation may be considered difficult for care givers [5]. Furthermore, it is a current opinion that hormonal therapy may be associated with possible side effects.

For these reasons, the care path for GHD is very demanding not only for the clinical condition but also for the psychological impact. The illness involves the whole family, not only the ill children, who during the years of therapy become adolescents, but also their parents and other members of the family like siblings and grandparents.

The team in charge of caring for children with GHD is composed of different roles: a core group represented by pediatricians, endocrinologists, psychologists and nurses, more other specialists on call such as nutritionists, diabetologists, cardiologists and geneticists. The teams are strongly involved in the care, with the important duty of following children through the whole pathway, from the diagnostic phase to the end of therapy, with continuous dedication.

The evolution of treatment, indeed, is varying. After the first 2 years of remarkable increase of height, generally the growth velocity declines over time, but it continues to be greater than would occur without treatment [6]; although this is a normal rate, the apparent loss of effect might be interpreted by children and their families as a loss of efficacy. They may become impatient to see faster and more effective results from therapy [7]. At this time, it is important to communicate that growth is a slow process that is measured over months, thus providing them with emotional support and new strategies of concordance to therapy. A reduced growth velocity may be due to a poor compliance to GH therapy in particular in pubertal patients [8]. An appropriate interaction between families and care teams can be achieved through the rigorous activity of listening and understanding evidence from those who live the condition of GHD and its daily implications. Narrative medicine permits this act of desired engrossed listening.

### Narrative medicine

Since 1999, the practice of Narrative Medicine has been defined by Greenhalgh and Hurwitz as “what is circumscribed between the physician and the patient, from the collection of information on events before the disease, to how it has been revealed, focusing on psychological, social and ontological implications” [9].

Narrative Medicine gives clinicians fresh methods with which to make contact with patients and to come to understand their points of view [10]. Collecting stories from patients, their families, healthcare and social professionals, permits attention to be given to how the person lives his/her illness, and gives meaning to the pathway to be carried out together with the healthcare team.

The difference between the two words ‘disease’ and ‘illness’ reflects the integration between Evidence-Based Medicine and Narrative Medicine; ‘disease’ may be intended to define the condition from the practitioner's perspective, considered as objective, as an alteration of the biological structure or functioning and mechanism of the body. The word ‘illness’, according to the definition given by the anthropologist Kleinman, means the human experience of the physical condition, including the person's perceptions, feelings and thoughts [11].

While the Evidence-Based Medicine cares for the monitoring clinical pathophysiological processes, Narrative Medicine includes the person's judgment when coping with distress caused by clinical problems. Narrative allows for the possibility of understanding the patient's and physician's real needs, which cannot be captured by questionnaires or any other means [12]. Through the narration form, patients and healthcare professionals’ deepest experiences, values and needs can be discovered.

Physicians who are studying and experimenting with Narrative Medicine, bring a testimony of how this approach changed their clinical practice, reducing anxiety and insecurities on both patients and professionals [13–15].

Furthermore, Narrative Medicine is a systemic approach to improve the organization and efficacy of healthcare services: “Narrative Medicine fortifies clinical practice with the narrative competence to recognize, absorb, metabolize, interpret and be moved by the stories of illness … helps doctors, nurses, social workers and therapists to improve the effectiveness of care by developing the capacity for attention, reflection, representation and affiliation with patients and colleagues” [16,17].

Narrative Medicine is proven to be a catalyzer for generating sustainability; not only does it give back a possible lost sense to healthcare providers, but also this tool fights waste and inappropriate activities, filtering the essential from these useless procedures [18].

For these reasons Narrative Medicine has been more and more used by scientific societies, healthcare facilities, patients and families’ associations, as a systemic approach to understand how to improve the organization of pathways, healthcare and social services.

### Objective

The aim of the project named CRESCERE, which in Italian literally means ‘to grow up’, was to understand and depict the children and teenagers’ illness with GHD and the other involved stakeholders’ experience.

For the first time, a narrative-based approach was used to collect testimonies from young patients with GHD, their parents, siblings and the healthcare providers who compose the care team from expert centers. The activity of collection of narratives was proposed to 80 young patients, their parents and a not defined number of their care providers.

Through narratives, those living with the illness and therapy were explored to:

Understand the perceptions regarding the management and organization of pathways;
Understand the real impact of this condition on family, social and school life;
Identify from the expressed needs and expectations, useful recommendations to improve the care services.

## Methods

Under the patronage of the Italian Society of Pediatrics Endocrinology and Diabetology (SIEDP) and the Italian Association of Families with subjects with a GHD (AfaDOC), from April to December 2013, 11 Italian Hospital Pediatric Endocrinologies experts in the diagnosis and treatment of GHD were actively involved in the project, on a voluntary basis, after a training course on Narrative Medicine. The expert centers were selected on the basis of their geographical location, to reach a homogeneous overview of the pathway all over Italy.

To obtain comparable narratives, only subjects with a diagnosis of isolated GHD were included in the project, both females and males, excluding patients with syndrome of turner and other pathological conditions. No specific time from diagnosis was requested.

### Tools for narratives collection

Considering the process of the story of illness, the Greenhalgh's methodology was applied, which identifies three main phases: ‘falling ill’, ‘being ill’, ‘getting better’ or ‘getting worse’. The narrative follows a determined plot that begins before diagnosis and continues through the clinical encounter, investigations, development of a management plan, treatment and resolution or progression of the disease [19]. Understanding narrative context of illness provides a framework for approaching patients’ problems holistically and may uncover diagnostic and therapeutic options.

The semistructured draft was used, since it was considered to be the most efficient way to guide the narration on specific, homogeneous and comparable topics, without losing the narrative form. For each target group, a specific plot was created and five different structured stories were respectively proposed, with the appropriate language to:

Children with GHD, 8–12 years old;
Adolescents with GHD, 13–17 years old;
Children and adolescents’ parents;
Children and adolescents’ siblings;
Care team of reference.

The foreseen plot for children and adolescents with GHD stimulated them to write about their family, school and social life, to describe their living with medical visits and therapy, together with their expectations for the future (Supplementary data 1: semi-structured plot addressed to children and adolescents with GHD).

The plot addressed to parents explored the pathway from first signals, through diagnosis, until the impact of this condition on the whole family's life and the coping strategies. Furthermore, parents were invited to write about their feelings, needs, fears and expectations for the future (Supplementary data 2: semi-structured plot addressed to children and adolescents’ parents).

The drafts for providers of care aimed to explore their motivations, living the relationships and communication with patients and families, together with their considerations regarding the organization and management of pathways (Supplementary data 3: semi-structured plot addressed to providers of care).

Healthcare professionals were also given an optional possibility to experiment with the parallel chart [20,21], free spaces like diaries, in which they wrote the story of care on a specific patient, their relationship between him/her and the family, their feelings and opinions about the compliance and the general management of that pathway. Diaries were analyzed separately from the semistructured plots, since their narrative-free form was not considered comparable with the semistructured format.

As writing directly in the centers of care was considered distracting and limited, both families and healthcare providers were invited to fill in the plot at home, in a moment of tranquillity, without interruptions.

### Ethic statement

Each narrative was collected upon written informed consent. In particular, two informed consents were addressed to families, respectively, for the approval to give their own testimony, in the case of adults, and for the parents’ approval to collect the testimony from their children with GHD, in the case of minors. In both informed consents, an introduction to the project was specified, together with the guarantee to respect the anonymity in analyzing narratives and reporting the results, as foreseen by the Italian Privacy Law [22].

Furthermore, at the beginning of the project, a system of code was defined and agreed with all the centers of care, to guarantee the families’ anonymity, but also to be able to know the narratives’ geographical origin.

### Analysis of narratives

Narratives were completely anonymous and the collected stories were analyzed through an integrated approach between quantitative and qualitative research, which foresees a qualitative interpretation of texts, along with the use of a specific software named NVivo.

The qualitative analysis was carried out in a blind session by two separate researchers, to avoid a subjective interpretation of texts; in case of conflict, a third researcher was involved.

This first analysis was implemented by the NVivo program, which facilitates a rigorous textual analysis of recurring words and semantic expressions, based on the approach of the Grounded Theory [23], which assumes to carry out the analysis without any previous hypothesis. This software represents a tool, which facilitates theory building from the data, allowing to generate substantive codes: words are clustered in nodes based on specific text search; information can be analyzed through word frequency, comparisons and logical correlations to create models, graphs or charts [24]. Data can be reconstructed to reflect a view of reality.

Using software in the data analysis process has been shown to add rigor to qualitative research, since it reduces human error, especially in cases of high volumes of texts. It is that a combination of both thought and computer-assisted methods is likely to achieve the best results; the quality, rigor and trustworthiness of the research is enhanced [25,26]. Along with a qualitative interpretation of narratives, a broader overview is achievable without losing the value of each individual testimony.

Adults’ narratives, both from parents and providers of care, were also classified on the basis of the style of narration, which combines content with used language and length of sentences. Three clusters were identified:

‘Disease-centered’ narratives: focused on the clinical and mechanicist point of view, with a technical language and harsh texts, which are not open to emotional considerations;
‘Illness-centered’ narratives: focused on living with a particular condition, with an open language and a flowing narration on emotional, familiar and social point of view;
‘Between disease and illness-centered’ narratives: containing both technical and emotional elements;

Specific analyses were carried out for the different target groups and all the results were compared and interpreted together.

## Results

During the life span of the project, a total amount of 182 narratives were collected (Table 1).

Results show that only 16.25% of young patients and 10% of the parents who were proposed to give their testimony did not provide researchers with their narratives, indicating a satisfactory engagement of the target groups.

All the received narrations followed the complete semistructured plot and were considered valid for the analysis.

Families’ narratives came mainly from the Italian northern regions (55%) and in an almost equal percentage from the center (33%) and south (38%) of Italy. Narrations were not collected equally from the 11 centers for three reasons:

The number of current cases of isolated GHD is variable;
The different reaction and availability of families to the proposal of writing their story;
The Italian phenomenon of healthcare migration from south to north Italy, for a supposed Nitin.

To maintain the anonymity, the names of centers were not reported.

Narration was appreciated by families, who through stories opened themselves up to their living with the condition, writing in a clear and direct way their needs and expectations. Physicians embraced as well the new possibility given by narration and revealed they were open to experiment with the parallel chart, as proved from their diaries, which were fully optional.

Also, the instrument of the semistructured plot was revealed to be the appropriate solution to obtain comparable evidence without losing the power of narration.

### Findings from children & adolescents with GHD

The collected testimonies from young patients were written by males in 56% of cases and by 60% of teenagers. No significative gender effects were reported regarding style and content of narratives, neither in their living with the GHD, nor toward clinical visits and therapy.

Table 2 shows the main results.

As shown on Table 2, children and adolescents mainly indicated that their height had not represented a particular social problem, without revealing any social stigma from their peers, since they were growing up: “After having started therapy I feel very good with my friends, and I live my life like everyone”; “now I feel good with my classmates because I am growing up”.

Discriminations were mentioned in a few cases: “I do not feel very comfortable with my schoolmates and friends, because they often offend me, underlining the difference between me and them”.

Despite the described general appreciation for healthcare professionals and therapy, differences between the two groups of age emerged with reference to pathway and therapy. Children wrote to appreciate medical visits, although their fear especially at the beginning of the pathway, describe them as calm with also funny moments: “I take pleasure in the medical visits, where I meet the doctor and nice nurses, they make me smile”; “I like coming here, everybody is good and nice”!

Adolescents reported a different and more bothered mood: “Medical visits here at the center are boring, especially during the day hospital”; “medical visits are very boring but I have to understand that they are useful to me”; “I have been coming to this Center for 10 years. I cannot say that medical visits delight me”.

There is also a different way of living therapy. Children reported to mainly suffer the pain of injection, but they shown to be willing to face the pain of the needle in order to ‘conquer’ centimeters, their most important objective and reason for satisfaction, since they felt already rewarded for their sacrifice: “Therapy makes me grow up. I am already taller than my deskmate”; “therapy is a little bit painful, but it makes me stronger”.

On the other hand, adolescents described feelings of impatience and boredom toward the long period of sacrifice: “I hope to finish therapy as soon as possible. I think that all this is positive for my growth but at the same time it is boring”; “I think therapy is helping me, but in the evening, I become discouraged”; “it is annoying doing therapy every evening”; “it is a constant task”; “I would like a weekly or better monthly drug, or at least to have a free day from therapy”.

Among the other results not represented in Table 2, the moment of communication of the diagnosis and therapy did not represent a traumatic phase and 26% of adolescents remembered to have been followed during this phase by their centers of care and families; finally, the future expectations were different of children and adolescents, as this last group expressed an overview of the future in a more explicit way linked to their growth and their desire to become taller (32% of adolescents): “For the future, I imagine to be tall enough and to not be different from other people anymore”; “in the future, I think I will be tall, powerful, proud of myself”.

### Findings from children & adolescents’ parents

In parents’ narrations, the story of pathway emerged in a more detailed way, from the beginning, when the first signals and suspects appeared, through the development of the phases during the years of therapy.

Parents were represented by 66% of mothers and 34% of fathers, without revealing remarkable differences in their living style and telling the experience of care. Narratives did not show differences depending on their children's gender.

Table 3 shows the main results from parents’ testimonies.

As shown on Table 3, most of the parents remarked the slowing down of their children's growth process on their own, usually during the first years of the kindergarten or the primary school, comparing them with their schoolmates and friends. But there was also a proportion of parents who reported a delay of the medical intervention because of the district pediatrician's underestimation of the problem: “I remarked problems in my son's growth immediately, but my pediatrician said to me that it was normal and I had to stay calm”.

Some parents told how they spent the period of waiting for diagnosis with anxiety and concern about the risk of an early hospitalization: “Waiting for the diagnosis caused anxiety in me, and I was scared about the beginning of treatment”; “I was worried, suspicious about so many medical exams for diagnosis (sample taking, cardiologic test, renal scan, endocrinologic visit, day hospital…)”.

Nevertheless, as well as for their children, the communication of the diagnosis did not represent a critical moment. The parents reported the physicians’ tendency since the beginning to prepare to the possibility of a GHD and reassure families about the existence of a therapy which is able to solve the problem. For these reasons, confirmation of the diagnosis was not a ‘traumatic surprise’.

With reference to the communication of the hormonal therapy, among the described feelings, some parents were worried and not completely convinced about therapy. Eventually, since they deemed this option was the only solution, they decided to follow the therapeutic project, but in their deepest thoughts they revealed being scared about the possible implications of hormonal administration: “When I knew about the existence of a care, a little bit of hope lighted up, even if I was not so confident”; “regarding treatment I am happy but at the same time hesitant”; “I have only some doubts regarding therapy”.

From the narratives, the hormonal injection was described mainly as an organizational issue, which requires a daily management to maintain the drug and can cause pain to their children. But 50% of parents were satisfied – “my son/daughter grows up well” – and 13% of them were largely satisfied – “my son/daughter grows up very well” – after the first results in their children's growth. ‘Well’ and ‘very well’ were one of the most common words used in the stories, to indicate the quality of services and the general satisfaction with therapy. Growth was their main aim and they were witnesses to the first successful outcomes; the value of the pathway was underlined in many testimonies: “Nobody makes fun of my son at school anymore”; “my son is more self-confident”; “he is growing up like his peers”.

Almost all the parents trusted the centers and the providers of care, but despite this general positive attitude toward the pathway, when asked about their worries, the issue of the consequences of therapy came out again: “I am worried about the possibility of developing a tumor”; “I wish therapy does not have future contraindications”; “I am wondering if my daughter will be able to have a child”; “I feel worried when I read the possible contraindications”.

The expressed doubts revealed a general confusion about knowledge on the physical consequences of the hormonal therapy. This means that although families were followed and empowered through the diagnosis and the beginning of the treatment, despite their satisfaction with the results and living with the daily injections without particular impacts, in their deepest thoughts they revealed to have persistent worries about side effects, sometimes wondering about their original decision. They did not feel reassured enough about the secondary features of treatment that might arise in the future. They were confident in the result of the growth, but were wondering whether there would be a price to pay for these results.

Concerning the style of narration, 48% of stories were classified as ‘illness-centered’, since narrations contained emotional considerations on the familiar and social impact caused by pathway. In total, 25% of narratives were clustered into the ‘disease-centered’ category, as the whole narration was written with technical language, using scientific words and expressions, texts were limited to a description of the clinical pathway, without opening up to more personal considerations. Finally, 27% of narratives were considered ‘between disease and illness-centered’, as texts alternated technical elements with more narrative parts in which reflections and feelings were sketched.

Narrations stimulated parents to open up or, at least, to try to do that.

### Findings from healthcare professionals who care for GHD

To complete the overview on pathway for GHD, a last comparison with the 19 providers of care's stories was done. This last point of view was composed by pediatrician endocrinologists, head physicians, postgraduate students, nurses and psychotherapists. To guarantee their anonymity, the professionals’ sources were not reported.

Table 4 shows the main results.

As shown in Table 4, professional experts revealed having a strong motivation, even those who had been working for >20 years, as highlighted also by the expressed metaphors: “I would describe my job through the figure of a gardener who cares for his little plants helping them to grow up”; “I feel like an athlete who unfailingly runs to improve his performance”.

From the narrations, they reported carrying out the medical visits for GHD in a constructive way, trying to transmit tranquility, availability and empathy to children and families, since the beginning of care. The communication of diagnosis was made with attention to the language used and aimed to reassure families.

Regarding therapy, their main concern was about its daily administration and correct management for a long time, the injection, the family's level of compliance, investigated in every follow-up. In total 53% of them reported being in difficulty when the failure of therapy caused disappointment of expectations.

Relationships with young patients were defined as an important condition to support them in the care. Particular attention was given to the relationship with children, considered the weakest subjects of the pathway: “I care in particular about children's expressions, what they do not say, their fears and hopes”; “I try to play with my little patients”; “I try to make children comfortable and place them at the center of my attention”.

Beside these testimonies of focused attention to support children at the beginning of the treatment, a few references to adolescents appeared, revealing a possible underestimation of care for their compliance and the relationship with them.

Relationships with patients’ families were indicated as fundamental to give the necessary support in fostering children during treatment. Nevertheless, compared with their patients’ narratives, the low frequency of the issue of the topic of side effects of therapy is remarkable.

Relationships with colleagues were considered important as well, a potential positive asset but still to be empowered by improving the exchange of knowledge and collaboration among the multidisciplinary care team.

Generally, they were satisfied with their working conditions and the quality of the offered services and they were positive toward the future.

In the case of care providers, the used style of narration was split between 39% of ‘illness-centered narratives’, in which emotional considerations appeared, 33% of ‘disease-centered narratives’ written with a technical language, as in a clinical chart and 28% of alternations between narration and professional descriptions. Although the illness-centered narratives prevailed, compared with families’ testimonies there was an increase in the disease-centered narrations. The dry and short texts revealed the reduced habit and possible embarrassment of writing in an unusual way, different from their daily clinical language.

### Physicians’ parallel charts

Although the 17 physicians’ diaries were separately analyzed, they represented an added value to the study, since they revealed important details on the management of relationships between physicians and families.

From parallel charts, the most important point of attention was the monitoring of both patients’ and parents’ compliance (41%), followed by the interest in social and school childrens’ lives (24%) and family dynamics (21%).

In these narratives, a more specific attention to adolescents’ level of autonomy appeared (14%), but they were considered still dependent on their parents in the management of therapy (69%).

A general tranquility was described with reference to the relationship between patients and physicians (29% both).

### Discussion & future perspectives

A first reflection regarding the number of collected narratives including the high proportion of children, adolescents (83.75%) and their parents (90%) who reported their narration, together with the optional testimonies from siblings and providers of care, can be considered an excellent result, which demonstrates the feasibility – and probably also the need – of the innovative narrative approach.

Healthcare professionals’ and patients’ narratives showed a reduced tendency to write their testimony and the request to describe their living with the illness could be considered quite unusual and strange. However, the high number of collected testimonies and the prevalent illness-centered style of narration revealed their enthusiasm and willingness to open themselves up to finding new ways of communication, interpretation and reflection on their care.

The 182 collected narratives can be joined to compose, through their recurring words and expressions, a ‘story of stories’, from which it is possible to identify possible recommendations on new strategies for pathways, communication, relationships and organization.

Analogies among the three points of view were more common than disagreements. Indeed, pathways of care were considered from all the subjects effective, both from the clinical and the organizational aspect.

The narrations were mainly stories of healing, although pathways were represented as long and demanding. Using Frank's Illness Narrative classification, the study reported stories of ‘restitution’, in which the plot involves returning to one's previous state of health [27,28]; after the suffering and the sacrifice, the victory comes and the treatment ‘repairs’ the body. In this specific case, the ‘conquest’ of an acceptable growth leads to well being and social inclusion.

Despite these common elements, different ways of living the care emerged with reference to therapy.

Teenagers underlined their impatience, up to the point of intolerance, toward the daily injection; they followed the pathway for years and, even if satisfied with their growth and still willing to become taller, showed signals of rebellion. This is probably connected to their age, but it is also indicative of the underestimation of the importance of their empowerment and active involvement in the pathway. Through their rebellion, they asked for another kind of relationship with parents and professionals, more focused on an autonomous role in the care. This attitude confirms the risk of a decrease in their adherence to therapy, as already shown in other scientific studies, in which it is demonstrated that adolescents have the highest rate of noncompliance or reveal discontinuation of GH treatment [29,30].

Parents can have an important task to help their kids to become more autonomous and responsible, changing their own role in the pathway and gradually decreasing their presence both in the ordinary administration of therapy and during medical visits. Empowering adolescents to be the main ones responsible for their care, probably can help them to find the strength and the willpower to go on with therapy, avoiding the risk of discontinuity or withdrawal.

As referred to by providers of care, they paid a lot of attention to children, especially at the beginning of treatment, to follow them through this particularly difficult and decisive phase of the care.

There is an increasing demand for physicians in pediatric settings to address not only the physical but also the psychosocial health of their adolescent patients, to prevent or intervene with health-risk behaviors and adhere to treatment recommendations [31,32]. Relationships addressed to children and their parents must change over the years, as children become teenagers and a new balance has to be found, adapting the pathway to the patients’ new needs and feelings. More attention should be paid to the adolescents’ ‘dilemma’ of willing more dedication on one hand and a more autonomous role on the other hand, helping them to achieve age-appropriate skills and feel adequate in case they are never as tall as they would like.

If centers wish to get a level of excellence, they should adopt specific policies for managing adolescents. The pediatric settings where they are visited are designed and adapted mainly for children, with the colors of the walls, furniture, toys and books. This is not what a teenager expects from a center of care: perhaps some centers that are more specifically designed to meet their tastes with different reading devices, e-devices, new spaces, together with a different language and consideration from care teams, could contribute to make them feel more understood. For instance, it has been shown that internet offers to adolescents the opportunity to give immediate computerized, tailored feedback on health and health behavior, in a more enjoyable and engaging way [33,34].

From parents’ narratives, 67% of them revealed having a persistent worry about possible and unknown side effects of therapy. They have lived since the beginning of treatment the conflict between the desire to offer to their children the solution of the growth problem and to ‘get’ a normal child, and their doubts regarding the hormonal ordinary and long term – almost chronic – administration. They asked for more reassurances about the consequences of therapy and their children's future, but this issue was a few mentioned in the healthcare's testimonies. In a long process, such as the treatment for GHD, which requires the need to be patient and to face several moments of discouragement, this hidden element can represent an alarm bell for the compliance to therapy. If families are not strongly confident in the treatment, they could decide to abandon the project of care, since results do not always appear from the very beginning.

However, parents reported accepting with particular devotion all the management of therapy; interestingly, nobody wrote about a possible failure of therapy and all of them trusted the effectiveness of the hormone, despite its possible side effects.

The healthcare teams can draw two considerations on relationships as narratives suggested the need:

To find a more engaging way of communication with adolescents, who require attention but in a different way compared with children.
To not neglect the families’ constant need for reassurance about therapy and its possible effects.

As providers of care revealed being aware of the importance of communication and empathy in this particular pathway, there is a fertile ground on which to work on, to improve relational skills, together with a high level of clinical competences.

It is possible to conclude that narratives revealed to be the right tool to allow all the family members to express themselves and provide health care professionals useful elements to understand how to improve their support, especially during particularly delicate phases, like adolescence.

**Table 1.** **Narratives collection according to origin.**

**Stakeholder**	**Number of stories**	**Female**	**Male**	**Percentage of collected stories^†^**
Patients with GHD	32 children, 35 adolescents	28	39	83.75%
Patients’ parents	72	45	22	90%
Patients’ siblings	7	5	2	no particular number was expected
Providers of care	19 stories 17 parallel charts	Not available	Not available	No particular number was expected

^†^The indicated percentages are the results of the number of people to whom the activity of narration was proposed and accepted to give their testimony, out of who did not accept.

GHD: Growth hormone deficiency.

**Table 2.** **Findings from children and adolescents with growth hormone deficiency.**

**Persons with GHD/topics**	**Children (8–12 years old)**	**Adolescents (13–17 years old)**
Social and school life	n = 34; “Very good:” 9 (26%); “Good:” 13 (38%); “Enough well:” 9 (26%); “I feel discriminated:” 3 (9%)	n = 43^†^; “Very good:” 13 (30%); “Good:” 18 (42%); “Enough well:” 8 (19%); “I feel discriminated:” 4 (9%)
Outcomes of GHD therapy	n = 20; “I am satisfied because I am growing up:” 11 (55%); “It is not so invasive:” 3 (15%); “I don't like it but I have to do it:” 4 (20%); “I don't like it at all:” 2 (10%)	n = 38; “I am satisfied because I am growing up:” 18 (47%); “It is not so invasive:” 4 (10%); “I don't like it but I have to do it:” 12 (32%); “I don't like it at all:” 4 (10%)
Living the medical visits	n = 20; “With tranquility and enthusiasm:” 8 (40%); “I feel scared:” 8 (40%); “with boredom and impatience:” 2 (10%); other: 2 (10%)	n = 18; “With tranquility and Enthusiasm:” 3 (16%); “I feel scared:” 1 (6%); With boredom and impatience: 7 (39%); other: 7 (39%)
Critical aspects in living the therapy	n = 28; “I suffer for the pain of injection:” 15 (54%); “I suffer for the daily task:” 7 (25%); “I cannot move from my house:” 2 (7%); “None critical aspects:” 4 (14%)	n = 34; “I suffer for the pain of injection:” 11 (32%); “I suffer for the daily task:” 17 (50%); “I cannot move from my house:” 4 (12%); “None critical aspects:” 2 (6%)

^†^The indicated numbers can imply more than one answer from each child/teenager.

GHD: Growth hormone deficiency.

**Table 3.** **Findings from children's and adolescent's parents.**

**Topics**	**Children and adolescents with GHD's parents**
The first signals of GHD	n = 65; “I remarked the slowing down by myself and looked for a center of care:” 35 (54%); “The pediatrician remarked the slowing down:” 23 (34%); “The pediatrician underestimated the slowing down:” 7 (11%)
The waiting for the diagnosis	n = 48; “Lived with anxiety and concern:” 26 (55%); “I had many doubts:” 3 (6%); “Lived with tranquility:” 17 (35%); “I was confident and optimistic:” 2 (4%)
The communication of the diagnosis	n = 48; “I was already prepared:” 18 (38%); “I was calm and confident:” 15 (31%); “I was released for the answer:” 1 (2%); “I felt normal:” 2 (4%); “I did not understand:” 3 (6%); “I was sad:” 4 (8%); “I was worried:” 5 (11%)
The communication of the therapy	n = 51; “I was worried and not convinced:” 16 (30%); “I was happy:” 15 (29%); “I was hoping:” 11 (22%); “I was released for the solution:” 9 (18%)
The difficulties of the therapy today	n = 89^a)^; “It is an organizational issue:” 29 (33%); “It is a painful task:” 19 (21%); “The medical visits are demanding:” 7 (8%); “I have to foster my son/daughter:” 6 (7%); “There are psychological consequences on my son/daughter:” 4 (4%); “It is long lasting:” 3 (3%); Other: 11 (13%); None difficulties: 10 (11%)
The outcomes of the therapy	n = 75; “My son/daughter grows up very well:” 10 (13%); “My son/daughter grows up well:” 37 (50%); “My son/daughter grows up enough well:” 7 (9%); “My son/daughter grows up a little:” 12 (16%); “My son/daughter grows up very little:” 1 (1%); Other: 8 (11%)
The centers and care teams	n = 56; “I totally trust the center and the team of care:” 38 (68%); “I trust the center and the team of care:” 17 (30%); “I trust the center and the team of care but not always:” 1 (2%)
Expressed fears and worries	n = 42; “I am worried about the side effects of the therapy:” 28 (67%); “I am afraid the therapy does not work:” 6 (14%); “I am afraid my son feels inferior:” 1 (2%); “I do not know/do not have worries:” 7 (17%)
Style of narration	n = 69^b)^; Disease-centered: 23 (25%); between disease/illness-centered: 24 (27%); Illness centered: 44 (48%)

^†^The indicated numbers can imply more than one answer from each parent.

^‡^In not all the stories were possible to use the classification of Kleinman.

GHD: Growth hormone deficiency.

**Table 4.** **Findings from healthcare professionals who care for growth hormone deficiency.**

**Topics**	**Healthcare providers**
The choice to work in pediatrics endocrinology	n = 17; Interest in pediatric growth problems: 7 (41%); passion: 6 (35%); other: 4 (24%)
What I try to transmit to patients during the medical visits	n = 30^a)^; Tranquillity: 14 (39%); my availability: 10 (31%); empathy: 5 (27%); information: 1 (3%)
During the communication of the diagnosis	n = 18; I pay attention to the language: 8 (52%); I focus on the solution of the treatment: 5 (31%); I use examples: 4 (13%); I give parents the choice: 1 (4%)
The most difficult issues in managing the therapy	The families’ daily task: 21 (34%); the injection: 12 (21%); the family's level of compliance: 8 (14%); the fear for the side effects: 7 (13%); the medical visits: 5 (9%); the anxiety for the result: 4 (7%); the individuation of possible problems: 1 (2%)
Relationships with patients	n = 19; Important: 8 (42%); particularly important with children: 6 (31%)
Relationships with patients’ families	n = 19; Important: 5 (26%)
Relationships with colleagues	n = 19; Important: 7 (37%); to be empowered: 5 (26%)
The quality of the offered services of care	n = 19; Satisfied: 15 (80%)
Style of narration	n = 18^b)^; Disease-centered: 6 (33%); between disease/illness-centered: 5 (28%); illness-centered: 7 (39%)

^†^The indicated numbers can imply more than one answer from each healthcare professional.

^‡^In not all the stories were possible to use the classification of Kleinman.

**Executive summary**

**Findings from children and adolescents with growth hormone deficiency**

Appreciation of healthcare professionals and satisfaction for the outcomes of therapy.
Adolescents show impatience toward pathway and the ordinary therapy.

**Findings from children and adolescents’ parents**

Appreciation of healthcare professionals and satisfaction for the outcomes of therapy.
Concern about possible unknown consequences of therapy.

**Findings from healthcare professionals who care for growth hormone deficiency**

Satisfaction for their working activity and conditions.
Attention to the children's adherence to therapy, but less remarked attention to the adolescents’ one.

**Future perspectives for providers of care's clinical practice**

To find a more engaging way of communication with adolescents, who require attention but in a different way compared with children.
To not neglect the families’ constant need for reassurance about therapy and its possible effects.
