# Supplementary material for: Stories of experiences of care for growth hormone deficiency: the CRESCERE project
Source: Future Sci OA. 2016 Feb 25;2(1):FSO82. doi: 10.4155/fso.15.82 (PMC5137913; doi:10.4155/fso.15.82)
Supplement: Supplementary file 1 [file fso-02-82-s1.docx]

*Appendix 1 - Semi-structured plot addressed to children and adolescents with GHD:*

| Children 8-12 years old | Adolescents 13-17 years old |
| --- | --- |
| Hi! Let’s talk about you, what do you do, what do you like, tell us about your family…  With your friends and schoolmates…  Tell us about your visits at the Center: who do you meet and how are the people you meet…  Tell us about when you get care at home: what do you like and what do you not like…  Let’s talk about you a little bit more: what would you like to do in the future… | Hi! Let’s talk about you, what do you do, what do you like, tell us about your family…  With your friends and schoolmates…  Tell us about your visits at the Center: who do you meet and how are the people you meet…  Your memories about the first visits at the Center, when you had to start the care: what did they explain to you, how did you feel…  Tell us about when you get care at home: what do you like and what don’t you like…  Let’s talk about you a little bit more: what would you like to do in the future… |

*Appendix 2 - Semi-structured plot addressed to children and adolescents’ parents:*

| **Role in the family**: Mother/Father  **The pathway to care**  How did we realize that there was something different in our son/daughter’s growth and when…  How did I feel during the waiting for the diagnosis, how long was the period…  The communication of the diagnosis…how did I feel when I knew about the existence of a therapy…  The beginning of the treatment…  How long did it take before I turned to this Center, the previous Centers…  Positive and critical elements of the pathway…  **The relationship with the equipe**  In what way did physicians communicate the state of my son/daughter’s growth, their words…the communication with my son/daughter…  My confidence in the equipe, how much do I feel reassured, my reference points…  **Living with the pathway**  How is my son/daughter growing up…  How do I feel when I administer therapy to my son/daughter…  How is my son/daughter living this experience of care…how is the family living the experience of care…  **The changes**  What has changed in me, in my emotional life, in my family, at work, in my social life…  Do I talk about the experience with somebody…the reasons…  What has changed in my son/daughter’s life…in the family, at school, with friends…  What has changed in the rest of the family…  What has changed in my partner’s life…  **Expectations, worries and hopes**  My expectations…my worries…my hopes…  What did I learn from this experience…  How do I imagine the future… |
| --- |

*Appendix 3* - *Semi-structured plot addressed to providers of care:*

| **My role in the equipe:**  **I have been doing this job for…**  **My choice to take care of growth**  The reasons why I chose to take care of growth…  If I used a metaphor to describe my job…  **The beginning of the pathway to care**  What do I try to transmit since the beginning to families and children…  How do I explain therapy to children and adolescents…to their parents…  The communication of diagnosis…for me, for patients and their families…  I always pay attention to…  **Criticalities in the pathway**  From the physician’s point of view…  From the patients’ point of view…  From the parents’ point of view…  In case of failure of therapy…  **The help for families**  The better way to support a family during a pathway for GHD…  What do I think about therapy and the care I can offer to patients…my organizational limits…  **The relationships**  The importance of the relationships with my colleagues…with children and adolescents…with their parents…  **Strong points and possible improvements**  My strong points…the equipe’ strong point  What would I like to improve…  The future… |
| --- |
